# Plantar vein thrombosis: a rare differential diagnosis in patients with plantar pain

**DOI:** 10.1590/0100-3984.2015.0075

**Published:** 2015

**Authors:** Maurício Fabro, Sara Raquel Madalosso Fabro, Rafael Santiago Oliveira Sales, Cesar Augusto Machado, Gustavo Lopes de Araújo

**Affiliations:** 1 Hospital Santa Catarina de Blumenau, Blumenau, SC, Brazil.

*Dear Editor,*

**Figure 1:**
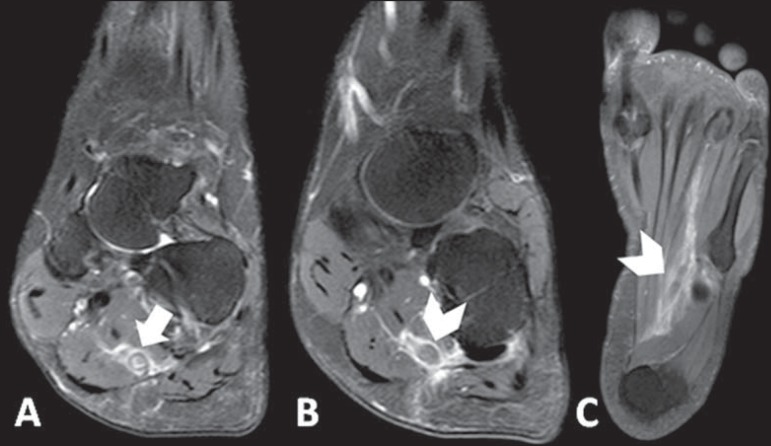
**A:** Proton density-weighted sequence with fat saturation demonstrating parietal thickening of lateral plantar vein in association with perivascular edema (arrow). **B,C:** Contrast enhanced T1-weighted sequence showing intraluminal filling defect and perivascular enhancement (arrowheads).

**Figure 2:**
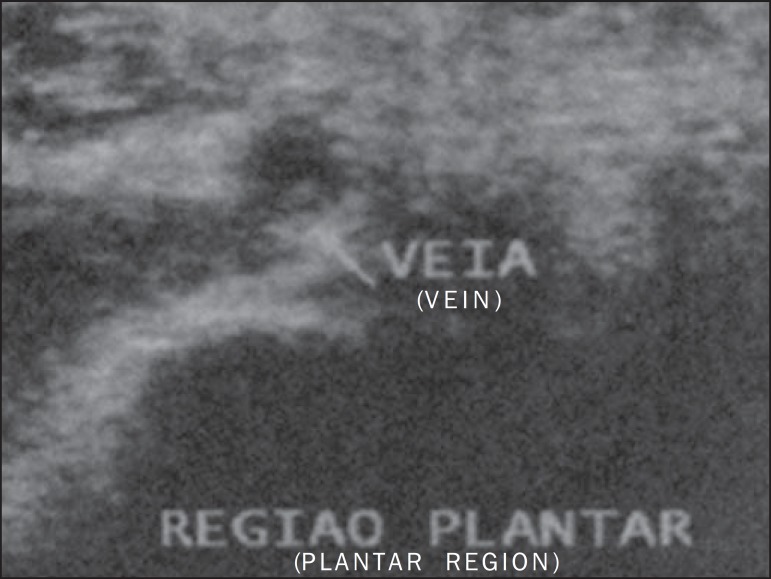
Ultrasonography of plantar region showing hypoechoic material within the lateral plantar vein in association with ectasia and non-compressibility of the vessel, as well as absence of flow at Doppler study.

A female, 42-year-old, smoking patient with previous history of thrombotic thrombocytopenic purpura, undergoing corticosteroid and plasmapheresis therapy was admitted to the hospital presenting with important pain in the plantar region of her right foot, with difficulty in ambulation starting about one week ago. The patient denied the occurrence of any trauma, previous surgery or recent travel. At physical examination, only hyperalgesia was observed at palpation. Magnetic resonance imaging (MRI) of the right foot demonstrated thickening and failure in filling in the whole extent of the lateral plantar vein associated with perivenular enhancement and edema of adjacent structures, suggesting the presence of plantar vein thrombosis (Figure 1). Supplementary ultrasonography showed the presence of hypoechogenic material within de lateral plantar vein in association with ectasia and non-compressibility of the vessel, as well as absence of flow at Doppler study - findings which corroborated the initial hypothesis (Figure 2). The patient was discharged with recommendations for rest and treatment with nonsteroidal anti-inflammatory drugs (NSAIDs), and presented with symptoms improvement at clinical follow-up.

Plantar vein thrombosis (PVT) is a rare condition^(1)^ characterized by development of an intraluminal thrombus in plantar veins, with less than 100 cases published in the literature^(2)^. It preferentially affects women of mean age 58.2 years^(3)^. Generally, the lateral plantar vein is most affected (in 96% of cases), followed by the middle plantar vein (in 41%)^(3)^. Several causal factors are speculated, among them traumas^(4,5)^, paraneoplastic syndromes^(6,7)^, postoperative conditions^(4,7)^, thrombophilias^(2,6)^, use of contraceptive drugs^(2,4)^, immobilization^(5,6)^ and HIV infection^(7)^. However, most cases are classified as being idiopathic^(2)^.

Cardinal signs and symptoms of PVT include recent onset^(7,8)^ of plantar pain^(1-3)^ and local edema with a typically unilateral presentation^(1)^ in association with limited ambulation^(2)^.

Considering the wide spectrum of differential diagnoses, the imaging diagnosis methods represent a useful tool for a correct characterization of the disease.

Ultrasonography is considered to be the main imaging method for the diagnosis of PVT^(1,3)^, demonstrating hypoechoic venous content^(2,6)^ associated with ectasia^(2,5,6)^, loss of vascular compressibility^(2,4)^ and absence of flow at Doppler study^(2,6)^. However, plantar veins are neglected at routine examinations^(1,2,4)^, which may explain the low rate of diagnosis of the disease.

MRI has gained prominence as besides making the diagnosis of PVT it can rule out other possible causes of plantar pain^(2)^. Main findings include characterization of intraluminal filling defect in plantar veins^(2,4,6)^ in association with edema^(5,6)^ and perivenular enhancement^(5)^. Such findings were observed in 100% of cases in one of the greatest studies approaching the theme^(5)^. On the other hand, only in 2008 the first case of PVT diagnosed at MRI was described^(9)^.

There is no defined treatment for PVT^(1,3)^, and possible treatment strategies include use of anticoagulant drugs^(1-4,6)^, NSAIDs^(1,3,6)^, elastic socks^(3,6)^ and rest^(6)^. However, the different therapies have shown similar results.

The most important complications of PVT include thrombosis extension into deep veins in the leg^(7)^ and occurrence of pulmonary embolism^(1)^.

Amongst the differential diagnosis of PVT, plantar fasciitis^(2,4,5)^, tendinous involvement^(3,5)^, bursitis^(5)^, Morton's neuroma^(4,5)^, stress fractures^(2,4,5)^, sesamoiditis^(5)^ and ganglion cysts^(5)^. No description of death associated with PVT is found in the literature.
